# Exploring the Presence and Impact of Advanced Nursing Roles in Care Homes and Charitable Organisations: An International Systematic Scoping Review

**DOI:** 10.1111/jan.70212

**Published:** 2025-09-16

**Authors:** Siobhán Kelly, Claire Pryor, Melanie Stephens, Vanessa Heaslip

**Affiliations:** ^1^ University of Salford Salford UK

**Keywords:** advanced practice, clinical nurse specialist, community care, systematic reviews and meta‐analyses, workforce issues

## Abstract

**Introduction:**

Investing in advanced nursing roles (AN) in social care is a strategic priority to address workforce challenges, create new career pathways, improve outcomes and future‐proof the sector. However, there is limited understanding of these roles globally. This systematic scoping review maps the international presence and impact of post‐qualification advanced practice roles for registered nurses (RNs) working in care homes and charitable organisations.

**Design:**

This review was conducted following the methodology established by the JBI and adhered to the PRISMA extension for scoping reviews checklist.

**Methods:**

Studies were included if (1) they included RNs working in care homes, charities or not‐for‐profit health centres, (2) the RN was in a specialist, enhanced or advanced practice role and (3) if role details were provided. Studies were limited to those published in the English language between 2014 and 2024. Evidence was gathered from a comprehensive search of electronic databases (CINAHL, MEDLINE, Scopus, PubMed and Web of Science), grey literature, relevant webpages, and reference lists. Expert consultations were also conducted. Eligible full texts were reviewed in Covidence software by two independent researchers.

**Results:**

The search yielded 575 records, and 89 were taken forward for full‐text screening. A total of 20 met the inclusion criteria: 19 were concerned with AN roles in care homes, and one focused on a charitable organisation. The majority of these studies (*n* = 12) were conducted in North America.

**Conclusion:**

The literature on AN roles in care homes and charitable organisations is notably sparse. Despite this, the available evidence highlights substantial benefits, including improved care quality, enhanced resident outcomes and positive impacts on team dynamics. This review identifies four key themes: scope of practice, positive impacts, influencing factors and barriers, which provide a framework for policymakers, healthcare leaders and educators to optimise the contributions of this group within the evolving global social care sector.

**Public or Patient Contribution:**

Not undertaken because of the nature of scoping reviews.

**Clinical Relevance:**

This review highlights the crucial role of advanced nurses in enhancing care quality, resident outcomes, and workforce sustainability in care homes and charitable organisations. The findings provide direction for policymakers and health and social care leaders to further develop the role of nursing in social care settings globally.

## 1 Introduction

Despite growing recognition of nursing's critical role in social care (Skills for Care 2019), evidence of the valuable contributions RNs make (Cornes and Manthorpe 2022), emergence of new educational pathways (QNI 2022) and knowledge surrounding advanced nursing roles remain limited. Although such roles are well‐established in some countries—particularly in the Global North (Fulton and Holly 2021)—a comprehensive international understanding is needed. This review aims to map the presence and impact of enhanced, specialist or advanced roles for RNs in care homes and charitable organisations across different countries; identify key features of these roles and highlight lessons and implications for the advanced practice nursing roles in the United Kingdom (UK) social care.

### 1.1 Background

Advanced nursing roles are well established in some countries, whereas in others they are still evolving. The United States (US) pioneered the Clinical Nurse Specialist (CNS) concept as early as the 1940s (Cannaby et al. 2020). The number of specialist roles, as well as the skills, competencies and complexity of such roles, has increased markedly over time, particularly in high‐income countries (Fulton and Holly 2021). In the US, ‘Advanced Practice Registered Nurse’ (APRN) is an umbrella term encompassing four categories: Clinical Nurse Specialist (CNS), Nurse Practitioner (NP), Certified Registered Nurse Anaesthetist (CRNA), and Certified Nurse Midwife (CNM) (International Council of Nurses 2020, 6). Other terms, including Advanced Practice Nurse (APN) and Clinical Nurse Consultant (CNC), are also used internationally (Fulton and Holly 2021). In the UK, these roles emerged in the 1970s and expanded in the 1990s, driven by external factors such as challenges in medical staffing (Castledine 2002). The most common UK titles are Nurse Practitioner (NP), Advanced Nurse Practitioner (ANP) or Advanced Nurse (AN) (Cannaby et al. 2020).

Although ANs are typically recognised for their advanced expertise, bridging the gap between clinical knowledge and person‐centred care (Chan and Holly 2021, 3), the international landscape is complex because of inconsistent definitions (Mohr and Coke 2018). Each country adapts titles, scopes of practice, and expectations differently. In the UK, there is currently no mandatory certification or regulatory framework governing advanced nursing roles, and their definitions often vary depending on the practice context or employer. Consequently, the scope of practice and educational requirements can be broad. Roles are thus primarily defined by the area of practice (e.g., a specific population or specialisation) rather than by a level of practice, such as advanced (Leary 2021).

Nurse specialist roles have been discussed in a wide range of contexts globally, such as cancer care (Kerr et al. 2021), neurology (Aspinal et al. 2012), HIV (Tunnicliff et al. 2013) and breast health (Harmer 2018). However, there is limited focus on these roles in social care settings. In the UK, ‘Adult Social Care (ASC)’ is the publicly funded provision of services, outside of National Health Service (NHS) settings, which aim to support people with care needs to maintain and enhance their independence, whilst living with mental or physical illness or impairment (Longo et al. 2023). This may be in a person's own home, in a residential setting such as a care home, or in a care home that provides onsite nursing services. However, funding models of long‐term care vary significantly. In the UK, for example, NHS funding covers care needs requiring an RN, whereas alternative sources like local authorities, charities or self‐funding cover needs without this requirement. Globally, Germany, Japan and the Netherlands utilise social insurance; Norway and Sweden offer universal coverage; England and New Zealand employ means‐tested systems; France combines national, regional and municipal approaches; Australia prioritises ‘aged’ care funding and Switzerland emphasises out‐of‐pocket payments, often through private assets and mandatory health insurance (Horstman et al. 2023).

In the UK, recent initiatives, such as the appointment of a Chief Nursing Officer for ASC, have highlighted the growing importance of nursing in the sector (Department of Health and Social Care 2020). Current reports have provided a valuable foundation for understanding the scope and role of RNs working in ASC, while also highlighting the limited evidence base regarding their contributions (Cornes and Manthorpe 2022). Important explorations into recruitment and retention have followed, revealing some of the complex workforce challenges the sector faces (Steils et al. 2024). Additionally, educational pathways are now available, including Specialist Practice Qualifications (SPQ), one being in ASC (Nursing and Midwifery Council 2022; QNI 2022).

Research focusing on advanced roles in social care is not only timely but globally relevant, as the landscape of social care nursing continues to evolve. Research has the potential to inform policy, practice, and education, contributing to a stronger and more sustainable social care workforce worldwide. Although a range of reviews have explored advanced nursing roles—addressing topics such as job satisfaction (Castledine 2002), capabilities (Hako et al. 2023), and identity (Mackavey et al. 2024)—none specifically examine the international scope of these roles and their impacts in social care settings. Gaining these insights is crucial for developing effective leaders and ensuring high‐quality care, particularly in the context of an ageing population (World Health Organisation (WHO) 2024) and the growing global demand for social care services (Joseph Roundtree Foundation 2024).

To address this, this review offers an original contribution by directly addressing the limited academic focus on advanced nursing roles in social care settings and the lack of knowledge regarding their contributions by examining the international landscape of post‐qualification enhanced, specialist or advanced practice roles for registered nurses in care homes and charitable organisations. It aims to map the presence and impact of these roles across diverse contexts to provide a global perspective on advanced nursing practice in social care settings, identify international best practices and lessons learned and generate insights to inform UK social care policy and practice.

A systematic scoping review was chosen, given that the topic has a limited and fragmented evidence base. Scoping reviews are ideal for mapping what is known and identifying gaps that can inform future research and policy development (Munn et al. 2018). In the context of post‐qualification education and career opportunities in this context, it is important to include grey literature. This type of literature—such as reports from regulatory bodies, professional associations, and other non‐academic sources—can provide valuable insights that may not be covered in traditional academic research, offering a more comprehensive view of the available evidence.

For clarity and consistency, this review will use the term ‘advanced’ nurses/nursing (AN), while acknowledging that definitions and terms vary across contexts (see Section 4 for further details). Similarly, the term ‘care homes’ will be utilised throughout, recognising the international variation in terminology for these facilities.

## 2 Design

This scoping review was conducted following the methodology established by the Joanna Briggs Institute (JBI) (Peters et al. 2024) and adhered to the PRISMA extension for scoping reviews checklist (Tricco et al. 2018). In line with this, all authors collaboratively developed and reached consensus on a pre‐defined protocol, which was registered within the Open Science Framework (registration number https://doi.org/10.17605/OSF.IO/AT7MQ, last updated October 16, 2024). Ethical approval was not required because the study analysed published documents without involving participants.

## 3 Materials and Methods

### 3.1 Eligibility Criteria

Although JBI typically recommends a PCC (Population, Concept and Context) framework for scoping reviews, we adopted PICOS (Population, Intervention, Context, Outcome and Study Design) because of the nature of the research question, which focuses on an intervention (AN roles) and outcome (their impact in social care). Care homes and charitable organisations were selected as the focus of this review to represent the diversity of social care settings internationally. Full details of the inclusion and exclusion criteria are provided in Table 1:

**TABLE 1:** Inclusion/exclusion.

	Inclusion	Exclusion
Population	Registered nurses (RNs) working in care homes, charitable organisations or not‐for‐profit health centres	Staff other than RNs working in care homes or charitable organisations RNs working in prisons or hospices RNs working outside of care homes or charitable organisations (e.g., hospitals) RN's working in the community/general practice or district nurses
Phenomenon/intervention of interest	Evidence on post‐qualification specialist, enhanced or advanced practice roles for registered nurses working in care homes, charitable organisations or not‐for‐profit health centres	Studies focused on pre‐registration roles Studies related to post‐qualification roles outside of care homes, charitable organisations or not‐for‐profit health centres
Context	Studies published in English Studies published within the last 10 years	Studies published more than 10 years ago Studies not published in English
Outcome	A comprehensive summary of these roles (e.g., pathways, qualifications, scope of practice, impact on service users and provision, barriers/facilitators and future trends)	Studies that fail to clearly define or describe the roles, even if they touch on related topics like nursing in general or workforce issues
Study design	Empirical research Reviews Evaluations RCTs Grey literature	Opinion pieces Case studies Editorials Anecdotal evidence Theses Conference papers Study protocols

### 3.2 Information Sources

A comprehensive search was conducted across multiple databases (CINAHL, MEDLINE, Scopus, PubMed and Web of Science) to ensure inclusion of relevant studies. Grey literature sources included Policy Commons (which has international coverage), Google Scholar, professional nursing organisations such as the Royal College of Nursing (RCN), NMC, QNI and other related organisations like the British Geriatrics Society (BGS). The National Institute for Health and Care Excellence (NICE), National Institute for Health and Care Research (NIHR) Evidence and the DHSC websites were also searched. Lateral search techniques included stakeholder engagement: consulting with experts such as Skills for Care, members of the UK Social Care Nursing Advisory Councils (SNACs) and senior nursing programme leads or directors. This was undertaken to ensure all relevant evidence, from all sources, was considered. Further, reference lists were checked, and the ‘cited by’ option in Google Scholar was used.

### 3.3 Search

Test searches were conducted in October 2024, with the search string refined in collaboration with reviewers 2 (V.H.) and 3 (C.P.) following consultations with an experienced research librarian. The full search commenced in November 2024, with reviewer 1 (S.K.) conducting the searches across the identified databases and sources. To ensure a comprehensive scope of international literature, it was essential to include accurate terminology for advanced roles. To this end, we referenced Chan and Holly (2021, 10), who provide an overview of globally recognised terms and titles. The final search string is provided in Table 2 below:

**TABLE 2:** Search string.

(nurs*) AND (“social care” OR “care home” OR “residential home” OR “nursing home” OR “retirement home” OR “long‐term care facility” OR “long term care facility” OR “voluntary organisation” OR “voluntary organisation” OR “non‐profit” OR “non‐profit” OR “not‐for‐profit” OR “not for profit” OR “charitable organisation” OR “charitable organisation” OR “NGO” OR “non‐governmental organisation” OR “non‐governmental organisation” OR “non‐governmental organisation” OR “non‐governmental organisation”) AND (“special* role” OR “enhanced role” OR “advanced role” OR “expanded role” OR “extended role” OR “advanced practice nurse” OR “advanced nurse practitioner” “advanced practice registered nurse” OR “clinical nurse consultant” OR “advanced nurse” OR “nurse specialist” OR “nurse consultant” OR “nurse clinician” OR “speciality nurse” OR “speciality clinical nurse” OR “senior specialist” OR “clinical specialist”)

### 3.4 Selection of Sources of Evidence

A two‐stage screening process was conducted using Covidence. Reviewers one and two independently screened the titles and abstracts of 319 papers and met to resolve any discrepancies. They then independently screened the full text of 89 papers, with a third reviewer consulted on two remaining disagreements. Twenty papers met the inclusion criteria for the review.

### 3.5 Data Charting Process

An extraction template was designed in Covidence in consultation with all three reviewers. To ensure consistency and identify any necessary modifications, reviewers one and three piloted the extraction form by independently extracting data from a subset of three studies. As a result of this, minor edits were made to the form (clarification of wording, adding fields and combining fields). Although reviewer two was available for consultation to reach consensus if needed, the pilot test revealed no discrepancies between reviewers one and three. Reviewer one then extracted data from the remaining 17 studies.

### 3.6 Data Items

Data was extracted on article characteristics (e.g., title, author(s), year of publication, aim of the paper), research design, role detail (e.g., title, definition and setting), role context (e.g., scope of practice, impact and barriers), and future considerations (research directions, limitations and wider considerations regarding specialist roles in this context). See Table S1 for characteristics of the studies and key findings:

### 3.7 Critical Appraisal of Evidence

The review utilised several critical appraisal tools to ensure the quality of the included studies. The Mixed Methods Appraisal Tool (MMAT) (Hong et al. 2019) was used to assess all empirical research. For the two review papers, the Critical Appraisal Skills Programme (CASP 2025) checklist was used to evaluate the quality of the review process. Grey literature was appraised using the Accuracy, Authority, Coverage, Objectivity, Date, Significance Checklist (AACODS) (Tyndall 2019).

### 3.8 Synthesis of Results

Following data charting, Reviewer one familiarised themselves with the data, which was then uploaded to NVivo 20. A narrative synthesis approach was employed. This process included three main steps:

- Data were coded in NVivo within predefined themes (such as roles, impact, and facilitators/barriers) that were determined on the basis of the research objectives.
- NVivo facilitated the comparison and contrast of findings across studies, within and across these predefined themes, helping to identify similarities, differences and overarching patterns.
- A comprehensive narrative was constructed, synthesising the coded data and comparative analysis to provide an overview of the roles and their characteristics. To ensure cohesive interpretation and narrative development, feedback was sought from reviewers 2 and 3 to confirm that the themes were clear, consistent, and logically grouped.

## 4 Results

### 4.1 Selection of Sources of Evidence

**FIGURE 1:**
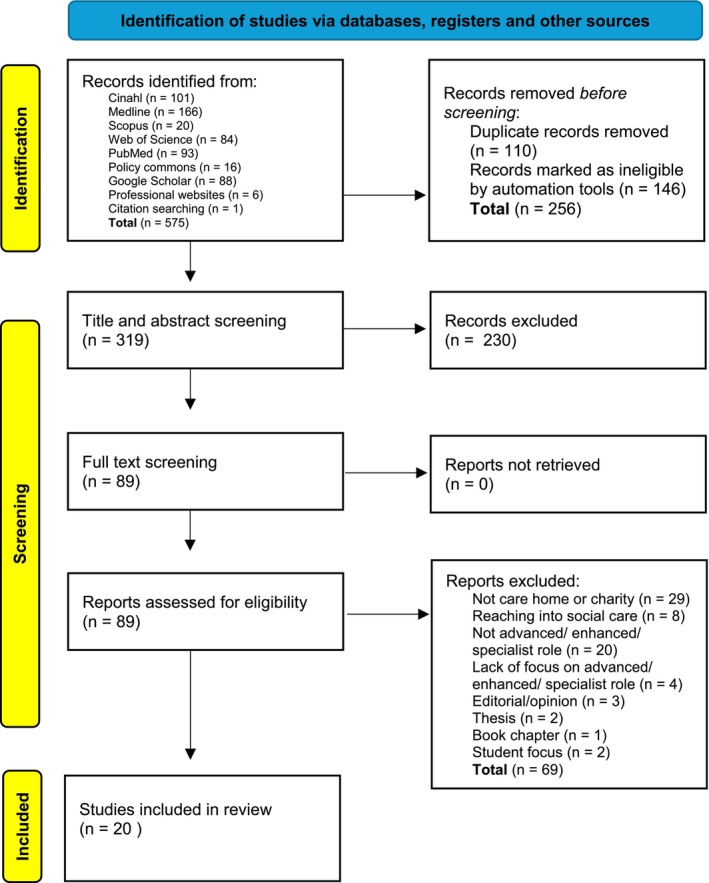
Selection process of papers for review (PRISMA 2021 flow diagram).

A total of 575 records were identified through searches across multiple sources, including databases and grey literature. After screening titles and abstracts, 89 studies were reviewed in full, and 20 were included in the final review. Studies were excluded primarily because of irrelevance to the research question. Figure 1 illustrates the selection process.

### 4.2 Characteristics of Sources of Evidence

This study uses the term AN for clarity and consistency. However, titles used within the included studies were diverse and reflected the geographic focus of the papers, with the majority centred on NPs. Table 3 below provides an overview of the advanced roles discussed in the studies:

**TABLE 3:** Advanced role titles.

Title	Studies
Nurse practitioner (NP)	Arendts et al. (2018); Campbell et al. (2020); Carpenter et al. (2024); Chavez et al. (2018); Alexander et al. (2023); Kilpatrick et al. (2020); Lovink et al. (2019); Lacny et al. (2016); McGilton et al. (2022)
Intercare nurse	Guerbaai et al. (2024)
Advanced practice registered nurse (APRN)	Flesner et al. (2019); Johnson and Harrison (2022); Kaasalainen et al. (2015)^a^; Lee et al. (2023)^a^; Rantz et al. (2017)^a^; Rantz et al. (2018); Lee et al. (2023)^a^
Registered nurse in expanded role (RNX)	Basinska et al. (2020); Favez et al. (2023); Saladino et al. (2024)
Admiral nurse	Bunn et al. (2016)

^a^ These studies utilised the term APRN to encompass both ANs and Clinical Nurse Specialists (CNSs).

The majority of the included papers originated from the US (Alexander et al. 2023; Johnson and Harrison 2022; Rantz et al. 2017, 2018; Carpenter et al. 2024; Flesner et al. 2019; Lee et al. 2023) and Canada (Campbell et al. 2020; Kilpatrick et al. 2020; Lacny et al. 2016; McGilton et al. 2022; Kaasalainen et al. 2015). European representation included Switzerland (Guerbaai et al. 2024; Favez et al. 2023; Basinska et al. 2021; Saladino et al. 2024) and the Netherlands (Lovink et al. 2019). One study originated from Australia (Arendts et al. 2018). Two reviews were also included: Chavez et al. (2018), which encompassed a broader international scope with papers from Canada, the Netherlands, Taiwan, and the US, and Bunn et al. (2016), which primarily focused on UK‐based literature with the addition of 11 systematic reviews.

Included studies represent a range of methodological approaches. The most common were quantitative studies (*n* = 9; Carpenter et al. 2024; Favez et al. 2023; Lee et al. 2023; Rantz et al. 2017, 2018; Saladino et al. 2024; Basinska et al. 2021; Lacny et al. 2016; Flesner et al. 2019), and qualitative studies (*n* = 6; Campbell et al. 2020; Alexander et al. 2023; Johnson and Harrison 2022; Kaasalainen et al. 2015; Lovink et al. 2019; McGilton et al. 2022). Further, one mixed‐methods study (Kilpatrick et al. 2020), one randomised controlled trial (Arendts et al. 2018), one report (Guerbaai et al. 2024) and two review papers (Bunn et al. 2016; Chavez et al. 2018) were also included.

All but one of the papers were set in care homes. The exception focused upon a charitable organisation (Dementia UK: Admiral Nurses) (Bunn et al. 2016). The emphasis of the papers can be broadly grouped into four categories: (1) Advanced roles and competencies: Studies examined how ANs provide care in care homes (Campbell et al. 2020), adapted organisational climate questionnaires for ANs in this setting (Alexander et al. 2023), summarised clinical settings and outcomes of advanced nursing care for older adults (Chavez et al. 2018), assessed the impact of AN roles on care quality (Kilpatrick et al. 2020), defined competencies and outcomes for ANs (Johnson and Harrison 2022; Basinska et al. 2021) and analysed the characteristics and activities of ANs (Saladino et al. 2024). (2) Models of care: Research evaluated coordinated AN care models (Arendts et al. 2018), explored initiatives to enhance care quality and reduce hospital transfers (Guerbaai et al. 2024) and assessed the cost‐effectiveness of an AN‐led model in long‐term care (Lacny et al. 2016). (3) Improving care practices: Studies investigated quality improvement projects in palliative care (Carpenter et al. 2024), examined ANs responses to evolving directives during the pandemic (McGilton et al. 2022) and reported on efforts to reduce antipsychotic medication use (Flesner et al. 2019; Rantz et al. 2017). (4) Factors influencing care: papers reported on the impact of ANs on quality measures (Rantz et al. 2018), explored AN engagement in quality improvement (Favez et al. 2023), identified causal links between AN activities and resident hospitalisation (Lee et al. 2023), analysed skill mix changes and their effects (Lovink et al. 2019) and highlighted the role of ANs as change champions in pain protocol implementation (Kaasalainen et al. 2015).

### 4.3 Critical Appraisal Within Sources of Evidence

Although the quality of individual studies was assessed using specific appraisal tools (see Section 3.7), this broader appraisal considers how well the collective body of evidence represented the topic area and whether the findings provided reliable insights for practice.

First, although some studies provided detailed definitions of advanced roles, the majority did not. For example, Campbell et al. (2020) described ANs as advanced practice nurses with a high level of expertise and autonomy in clinical practice, highlighting their role in elevating nursing practice. However, many studies (Kilpatrick et al. 2020; Rantz et al. 2017, 2018; Lovink et al. 2019; Lacny et al. 2016; Flesner et al. 2019; Lee et al. 2023; Carpenter et al. 2024) did not provide explicit definitions of the advanced roles under investigation. This lack of clarity, likely reflecting the assumption that the title is already well established and defined, can create challenges in understanding the precise functions and scope of these roles across the broader literature in this context.

Further, the majority of studies specifically focused on the roles, responsibilities, and impact of ANs, but others referenced the role within a broader mode of care, such as integrated care programmes or interprofessional teams. Guerbaai et al. (2024), for example, spoke about the INTERCARE (advanced) nurse within the context of a six‐component INTERCARE model. Similarly, Lovink et al. (2019) and Lacny et al. (2016) explored models of care involving ANs in conjunction with physician assistants or family physicians. This broader lens, though valuable for understanding the context of advanced nursing practice, may potentially dilute the findings specific to the nurses' roles and contributions. This variation in focus thus needs to be considered when interpreting and synthesising the evidence on the effectiveness and impact of ANs in social care.

It should also be highlighted that Bunn et al.'s (2016) paper was divided into two sections: (1) a systematic review of literature examining the scope and effectiveness of Admiral Nurses, and (2) a review of reviews focused on interventions designed to support family carers of people with dementia. However, data related to interventions in the second section could not be drawn upon. Although some had elements of role alignment with Admiral Nurses, either partially or wholly, they provided limited information about who delivered the evaluated interventions (a limitation that Bunn et al. acknowledged). As a result, little evidence could be drawn about the specific role and impact of Admiral Nurses working in care homes or charitable organisations in this paper.

### 4.4 Results

Four main themes were constructed from the data, all of which included three or four subthemes. The overarching themes are (1) Scope of practice, (2) Positive impacts, (3) Influencing factors, and (4) Barriers.

#### 4.4.1 Scope of Practice

The scope of practice for ANs was reported to be broad, dynamic and context‐dependent. The role was commonly centred around promoting and enacting change, with ANs described as change agents or champions to influence care practices and outcomes (Kaasalainen et al. 2015). However, the breadth of the advanced role also reflects its diversity, as it could vary significantly across different settings. For example, their scope could be expansive, encompassing a wide range of clinical responsibilities (Chavez et al. 2018), or described as looser and more autonomous, with ANs taking on independent decision‐making authority (Arendts et al. 2018). The variability in the role is further highlighted by the fact that the specific responsibilities and impact of an AN could differ depending on the local context and organisational needs, as seen in initiatives like those discussed by Guerbaai et al. (2024).

##### 4.4.1.1 Educating, Coaching and Leadership

Many papers emphasised education, coaching and leadership as key facets of the AN role. Saladino et al. (2024) reported 65.4% of participants had a leadership role, often influencing organisational practices and driving system improvements. Rantz et al. (2018; 2017) described ANs developing interventions to reduce acute care transfers, whereas Campbell et al. (2020) discussed ANs leading complex resident situations. Guerbaai et al. (2024) illustrated this leadership in ANs driving the implementation of comprehensive geriatric assessment, advance care planning and analysing data to optimise care quality. Coaching and support were also key. Basinska et al. (2021) reported ANs coaching and empowering staff, whereas Lovink et al. (2019) illustrated ANs supporting care teams through accessibility, coaching, education and understanding daily practice. McGilton et al. (2022) noted ANs emotional support, whereas Rantz et al. (2018) described ANs coaching staff and role‐modelling advanced assessment skills. Kaasalainen et al. (2015) further emphasised ANs encouraging staff to adopt evidence‐based innovations. Education was also crucial: McGilton et al. (2022) and Saladino et al. (2024) showed ANs educating other providers and consulting with staff to improve care. Further, Saladino et al. (2024) found 80.4% of ANs offered team education, and Favez et al. (2023) reported 87.5% involved in clinical teaching. Campbell et al. (2020) noted ANs providing palliative care education, and Arendts et al. (2018) described their remit educating families on diagnosis and prognosis. Although Basinska et al. (2021) suggested limiting AN involvement in direct resident education to complex situations, other studies, including Rantz et al. (2017) and Kaasalainen et al. (2015), supported ANs educating the wider team generally.

##### 4.4.1.2 Daily Provision of Care

The daily provision of care provided by ANs encompassed a wide range of activities, from following predefined protocols to complex responsibilities requiring advanced clinical judgement (Lovink et al. 2019). Johnson and Harrison (2022) emphasised the collaborative nature of this care, with ANs participating in interprofessional meetings (Lacny et al. 2016) and collaborating with physicians (Guerbaai et al. 2024; Lovink et al. 2019). *Direct* care activities included assessments (Flesner et al. 2019; Basinska et al. 2021), diagnosis, treatment (Campbell et al. 2020), medical rounds (Lovink et al. 2019), responsibility for subacute, chronic, and unplanned acute care (Arendts et al. 2018) and ongoing chronic illness care (Kilpatrick et al. 2020). Plus, proactive advance care planning (Basinska et al. 2021) and running family meetings (Lovink et al. 2019; Lacny et al. 2016; Carpenter et al. 2024) were also apparent. *Indirect* clinical activities included documentation, consultation, medication reconciliation, medication review and supporting deprescribing (Campbell et al. 2020; Kilpatrick et al. 2020). Although Basinska et al. (2021) focused on the AN role as being centred around medication monitoring rather than prescribing, Lovink et al. (2019) and Lacny et al. (2016) indicated that ANs did prescribe medications. Further, Arendts et al. (2018) described ANs liaising with primary care doctors, coordinating care and utilising best practice guidelines. ANs addressed a variety of clinical issues, with Carpenter et al. (2024) finding that all ANs addressed pain, whereas many addressed dyspnoea, delirium, weight loss, congestive heart failure and chronic obstructive pulmonary disease. Care for people living with dementia was particularly common, with Saladino et al. (2024) reporting it as a prevalent clinical focus of the AN workload. Bunn et al. (2016) provided more detailed insights into dementia care, describing the provision of emotional and psychosocial support to family carers, education about dementia and noted eight core competencies for Admiral Nurses, including therapeutic work, advanced assessment and balancing the needs of the carer and person with dementia. Palliative care also emerged as a significant aspect of specialist practice in care homes. Saladino et al. (2024) found that nearly half (44.2%) of ANs provided palliative care. Although Campbell et al. (2020) described ANs supporting patients with palliative care needs and Arendts et al. (2018) highlighted palliative care planning as central to the role, Carpenter et al. (2024) provided the most comprehensive overview: they described in detail ANs conducting palliative care visits, identifying residents at high risk for palliative care needs, making hospice referrals, and coordinating end‐of‐life care.

##### 4.4.1.3 Less Reported Aspects of Practice

Beyond the core aspects of AN practice, the literature revealed additional, less frequently reported, elements that contribute to their diverse scope of practice (Chavez et al. 2018). These included professional networking (Basinska et al. 2021) and navigating financial aspects of care—including legal and practice concerns around pay and financial restrictions (Alexander et al. 2023). Some studies also touched upon responsibilities related to research, workforce and policy. For example, Favez et al. (2023) found that just over a third (35.6%) of ANs had a research or innovation‐focused role. Similarly, Johnson and Harrison (2022) described ANs collecting and synthesising data for quality improvement initiatives and staff education, whereas Basinska et al. (2021) noted their involvement in developing guidelines and searching for evidence‐based practice. Guerbaai et al. (2024) demonstrated that the COVID‐19 pandemic further illustrated the adaptable nature of the AN role, with ANs taking on a range of new responsibilities around infection control, testing, isolation and guideline development. McGilton et al. (2022) similarly discussed ANs leading vaccine rollouts, ensuring protocols were followed, providing emotional support and collaborating with staff to implement new systems for observation, medication review and care allocation.

#### 4.4.2 Positive Impacts

The significant and far‐reaching impact of ANs in care homes and charitable organisations was well documented, with evidence highlighting their role in delivering high‐quality care, improving outcomes, strengthening team dynamics, and supporting residents, carers and families.

##### 4.4.2.1 High Quality Care

Evidence consistently demonstrated the crucial role of ANs in fostering high‐quality care within care homes. The very presence of ANs was described to signal a commitment to high‐quality care (Saladino et al. 2024), which is further evidenced by Lee et al. (2023), who found a causal link between AN consultations and subsequent RN consultations. Other studies reinforced this point: Lovink et al. (2019) emphasised the significant contributions of ANs to improved care quality, whereas Lacny et al. (2016) demonstrated quality enhancements achieved through an AN‐family physician model of care. A key benefit associated with the AN role was timely access to care, as highlighted by Campbell et al. (2020). Moreover, Basinska et al. (2021) and McGilton et al. (2022) demonstrated how ANs fostered person‐centred approaches that prioritised resident preferences and needs. Continuity of care was another prominent theme, with Chavez et al. (2018) and Lovink et al. (2019) demonstrating how ANs work hard to ensure consistent and cohesive care delivery. Finally, ANs frequently led and contributed to quality improvement initiatives, as evidenced by studies from Lovink et al. (2019), Rantz et al. (2018), and Favez et al. (2023), which documented their involvement in projects designed to enhance care delivery and resident outcomes.

##### 4.4.2.2 Outcomes

Resident outcomes were improved following care provided by ANs. Across the studies, reductions in hospital transfers and acute care utilisation were commonly reported (Campbell et al. 2020; Kilpatrick et al. 2020; Guerbaai et al. 2024; Rantz et al. 2017, 2018; Lacny et al. 2016; Lee et al. 2023). This was attributed to several elements such as advanced care planning (Guerbaai et al. 2024) and better management of health conditions (Rantz et al. 2017; Lee et al. 2023). However, not all studies showed significant reductions in transfers (Arendts et al. 2018). Beyond transfer rates, the transition process to acute care itself improved, with less strain on residents, especially those with cognitive impairments (Campbell et al. 2020; Rantz et al. 2018). Specific interventions yielded positive results, including fewer incidents of restraint and pressure ulcers (Kilpatrick et al. 2020; Rantz et al. 2018), improved medication management and better antipsychotic prescribing, potentially increasing engagement in daily activities (Flesner et al. 2019; Kilpatrick et al. 2020). Wider impacts included reduced staff turnover, strengthening of the nursing profession (Basinska et al. 2021), and the potential to address physician shortages (Lee et al. 2023). Financial implications were mixed. Although some found cost was not consistently improved (Chavez et al. 2018), others suggested potential cost offsets (Campbell et al. 2020; Rantz et al. 2018; Lacny et al. 2016), though cost‐effectiveness was also cautioned to require careful contextual analysis (Lacny et al. 2016).

##### 4.4.2.3 Team Dynamics

Improved team dynamics, with a strong emphasis on collaboration and integrated working, also emerged as a key impact. Kilpatrick et al. (2020) highlighted the positive impact of multidisciplinary collaboration involving ANs on care models and resident outcomes, whereas Arendts et al. (2018) emphasised the importance of integrated care models, which involve ANs and are crucial for enhancing the quality of care. Chavez et al. (2018) provide an example in noting that collaborative models involving ANs achieved outcomes that were better than or equal to physician care alone. Also, the presence of ANs fostered several positive changes in team dynamics. Campbell et al. (2020) observed more collaborative interprofessional practice, whereas Alexander et al. (2023) reported improved relationships between staff and nursing directors. Carpenter et al. (2024) found that staff felt better prepared to care for residents with complex needs, and McGilton et al. (2022) noted increased confidence among staff members. Enhanced collaboration between physicians and ANs was also documented (McGilton et al. 2022). Further, Lee et al. (2023) found that embedded ANs facilitated increased communication and collaboration among care providers, particularly when changes in a resident's status occurred, and Kaasalainen et al. (2015) described how ANs involved the wider team in training and activities, fostering shared values and community engagement. Lee et al. (2023) also highlighted the active involvement of ANs in decision‐making processes, demonstrating their integral role within the multi‐disciplinary team.

##### 4.4.2.4 Residents, Carers and Families

Although no studies specifically focused on the perspectives of residents or families, some reported outcomes were relevant to these groups. ANs contributed to improved outcomes for residents in several ways, including increased resident satisfaction (Guerbaai et al. 2024) empowerment (Basinska et al. 2021) and improvements in residents' quality of life (McGilton et al. 2022; Arendts et al. 2018; Flesner et al. 2019). Positive outcomes for families and carers related to improved communication and shared decision‐making, with ANs facilitating more frequent meetings to keep families informed and promote understanding (Carpenter et al. 2024). Guerbaai et al. (2024) also reported increased satisfaction among caregivers. Bunn et al. (2016), the only paper included to specifically focus on carers, described in detail the ways in which ANs offer practical support to carers, such as assistance with accessing services, which led to high levels of carer satisfaction.

#### 4.4.3 Influencing Factors

This section explores the factors that influence the ANs' role in care homes and charities, ultimately shaping their integration and the positive outcomes achieved. As highlighted by Lovink et al. (2019), these influences operate at social, organisational, and professional levels.

##### 4.4.3.1 Assimilation and Autonomy

The assimilation of ANs into care home environments, and their autonomy within those settings is crucial for their effectiveness. Transitioning into these environments often involved leveraging prior experience and professional preparation. Saladino et al. (2024) found that RNs were more likely to apply research findings, highlighting the value of master's‐level education. However, Johnson and Harrison (2022) observed that even experienced ANs initially focused on establishing credibility through basic nursing skills, positioning themselves as embedded caretakers to build goodwill among staff. Plus, integrating seamlessly into existing systems could facilitate effective practice. Kaasalainen et al. (2015) described the successful implementation of an AN‐led pain protocol by aligning it with the (long) established SBAR (Situation, Background, Assessment, and Recommendation) process in long‐term care facilities. Further, Basinska et al. (2021) emphasised the crucial role of autonomy in maximising the contributions of ANs, and Kilpatrick et al. (2020) noted the value of decision‐making autonomy within a consultative model of care (whereby ANs independently manage resident care within their expertise). In this vein, Johnson and Harrison (2022) found that access to patients and dedicated practice spaces facilitated smoother transitions and contributed to greater success for APRNs. Lovink et al. (2019) also observed that ANs and RNs exercised autonomy by focusing on the nursing domain, differentiating themselves from Emergency Care Practitioners (ECPs). This autonomy was found to enable them to prioritise patient‐centred care, build strong relationships and take the time to understand individual needs.

##### 4.4.3.2 Collaboration, Trust and Support

Where better collaboration is an outcome of AN engagement, it can also be understood as a facilitator of their impact. Collaboration, trust and support all emerged as key factors enabling this group's integration and effectiveness. Alexander et al. (2023) emphasised the importance of ANs building strong relationships with staff, whereas Campbell et al. (2020) highlighted collaboration as a cornerstone of successful AN practice. Kilpatrick et al. (2020) similarly observed that greater collaboration with pharmacists, alongside regular medication monitoring and autonomous decision‐making, led to significant decreases in the average number of medications prescribed to residents. This collaborative environment was further strengthened by supportive systems. Alexander et al. (2023) argued that simply adding ANs to the workforce is insufficient; rather, a supportive and collaborative care environment is essential for them to thrive and provide high‐quality care. Support from the director of nursing was identified as a key factor (Alexander et al. 2023). Also, Guerbaai et al. (2024) highlighted the importance of reciprocal learning, supportive leadership and organisational readiness for change. Rantz et al. (2017) provided an example, describing how the project intervention led worked closely with social services, primary care providers, nursing staff, and ANs to provide wrap‐around support and thus establish effective communication systems regarding residents' advance care directives. Kaasalainen et al. (2015) further emphasised that ANs often drive practice improvements by addressing barriers to change and supporting staff. They highlighted the importance of positive relationships, stating that successful change champions must be well‐connected, respected and trusted in their expert role. This emphasis on trust is echoed by Lovink et al. (2019), who found that trust and personal connections between ECPs and ANs were often considered more important than formal agreements.

##### 4.4.3.3 Vision and Commitment

The success of ANs in care homes and charities relies on both individual and organisation‐level factors. Alexander et al. (2023) highlighted the importance of individual commitment and passion for the role, echoing Guerbaai et al.'s (2024) note that a strong commitment to reducing hospitalisations contributed to the success of their model. Kaasalainen et al. (2015) found that ANs who were committed to—and persevered with—implementing new initiatives were crucial facilitators. Carpenter et al. (2024) also emphasised the value of committed mentorship from specialist champions to support ANs. However, individual commitment thrived within a supportive organisational context, which was reported to begin with acceptance by the care home team and wider community. Lovink et al. (2019) found that acceptance of ANs in care homes was positively correlated with familiarity with their expanded roles and responsibilities among managers, other healthcare providers and residents/families. Beyond individual commitment, organisational support for ANs' roles was understood to be essential, though not always a given. Lovink et al. (2019) observed that managers and ECPs often struggled to prioritise a shared vision for these roles because of the high pressures within nursing homes. Similarly, Carpenter et al. (2024) noted that organisational commitment to AN‐led interventions depended on staff capacity and their willingness to identify residents who may benefit. Flesner et al. (2019), in addition, found that leaders were more likely to support such roles after they had witnessed tangible positive impacts, such as residents experiencing benefits from reduced antipsychotic medication. Crucially, then, organisational readiness for change, including a willingness to embrace interprofessional collaboration, was essential for creating an environment where ANs can effectively implement their expertise (Guerbaai et al. 2024).

##### 4.4.3.4 Supervision and Surrounding Structures

Structural elements and robust supervision were critical factors in optimising the effectiveness of ANs in these settings. Rantz et al. (2017) highlighted the importance of regular supervision with oversight and guidance, reporting systems that enable performance monitoring and leadership meetings that foster engagement and direction. These mechanisms provided a framework for accountability, continuous improvement, and communication, ultimately enhancing the quality‐of‐care ANs provide. This is echoed by Kaasalainen et al. (2015), who advocate for a multifaceted approach that utilises educational outreach, audits and feedback to support successful interventions. Guerbaai et al. (2024) similarly emphasised the value of structured interventions, demonstrating how a multifaceted approach that includes leadership meetings, ongoing support and coaching sessions can effectively implement new models of care. Further, the implementation of policies, such as advanced directives, was found to ensure the systematic involvement of skilled professionals in complex care decisions (Lee et al. 2023). Flesner et al. (2019) add another dimension here, highlighting how structured programmes like Dementia Care Mapping (DCM) can increase ANs understanding of the needs of people with dementia, further equipping them to provide effective care. Effective supervision also played a crucial role. Guerbaai et al. (2024) demonstrated that reflection meetings, coupled with onsite clinical examinations by physicians or specialists, linked to the AN role to significantly reduce unplanned hospital transfers. Furthermore, Saladino et al. (2024) demonstrated that the type of supervision ANs receive influences their practice. For instance, those supervised by administrators were less likely to apply research findings or contact physicians than those supervised by directors of nursing or ward managers.

#### 4.4.4 Barriers

ANs, though making significant contributions, encountered various challenges in care homes and charitable settings that could hinder their integration and impact. These barriers, as identified by Alexander et al.  (2023), existed at both the individual and institutional levels, spanning a range of elements such as difficulties with documentation retrieval, burnout, heavy workloads, staffing shortages and legal and practice concerns.

##### 4.4.4.1 Institutional and Policy Level

Institutional and policy‐level factors could significantly impede the ability of ANs to effectively practise and achieve positive outcomes within the care home environment. Such barriers were reported to include issues with role regulation, title protection and funding models. Chavez et al. (2018) demonstrate how the lack of clear definitions and standardised titles for AN roles creates confusion and hinders integration, and variations in educational requirements and role titles across health and care systems were noted to further exacerbate this ambiguity. Financial constraints were another major obstacle. Rantz et al. (2018) identified restrictive US Medicare billing practices as an important barrier, noting discrepancies between regulations for ANs employed by nursing facilities versus those who are not. These limitations hinder care homes' ability to hire ANs and restrict resident access to their services. Similarly, Favez et al. (2023) highlight financial and institutional barriers in Switzerland and other countries, including challenges with education, recognition, and remuneration, which limit the expansion of AN roles in geriatric care. Poor recognition of advanced roles within care homes was also described as a structural barrier. McGilton et al. (2022) described that AN roles are often ‘hidden’ within the workforce, a systemic problem that undermines professional autonomy and limits their ability to contribute effectively. Johnson and Harrison (2022) provide an example of this, describing how preexisting care teams worked to exclude an AN from accessing patients. External policies and pressures could also challenge this group's effectiveness. McGilton et al. (2022) observed that resident‐centred practices were often compromised by levels of policies which prioritise safety over welfare. In addition, Bunn et al. (2016) highlighted how deficiencies in other statutory services, such as social care, increase the demands placed on ANs like Admiral Nurses, stretching their capacity and limiting their effectiveness. Arendts et al. (2018) also emphasised the complexity of emergency department transfer decisions, calling for policy‐level interventions to streamline these processes and ensure timely access to appropriate care.

##### 4.4.4.2 Role Ambiguity, Resistance and Conflict

Advanced roles were also hampered by a complex interplay of role ambiguity, resistance and conflict. Central to these issues was a lack of recognition (Bunn et al. 2016), clarity and understanding of the AN role (McGilton et al. 2022). Basinska et al. (2021) noted that poorly defined distinctions between ANs and RNs could fuel opposition and hinder the integration of ANs within care teams. Such ambiguity also created uncertainty and anxiety among existing staff, leading to perceptions of role overlap and threats to professional autonomy (Kaasalainen et al. 2015). This lack of clarity often resulted in misunderstandings and conflict, as illustrated by Johnson and Harrison (2022), who documented instances of direct conflict between ANs and medical directors, which sometimes required mediation. Rantz et al. (2017) further highlighted frustrations expressed by nursing home staff regarding physicians who transferred residents to the emergency room or hospital, potentially undermining the AN's clinical judgement. Campbell et al. (2020) acknowledged additional sources of conflict, including leadership disputes and pay equity concerns, which they linked to traditional hierarchical roles. However, they also suggested that fostering a collaborative approach could mitigate such resistance. However, conflict was not limited to interactions within care teams. Bunn et al. (2016), for example, found that Admiral Nurses frequently navigated complex family dynamics and had to balance differing viewpoints between carers and the person with dementia.

##### 4.4.4.3 Turnover and Time

High turnover rates among staff and leadership, coupled with time constraints and increased workloads, also posed significant challenges for ANs in this context. Kaasalainen et al. (2015) and Guerbaai et al. (2024) identified staff turnover as a key barrier to implementing and sustaining AN‐led initiatives. Rantz et al. (2017) found that care homes with higher hospitalisation rates also had greater leadership instability, disrupting continuity and impeding quality improvement. Further, Flesner et al. (2019) highlighted how staff turnover necessitates continuous orientation for new staff and can, in some cases, lead to the discontinuation of successful initiatives. Time constraints and increased workloads were also found to further hinder AN effectiveness (McGilton et al. 2022; Kaasalainen et al. 2015) as they could limit capacity to implement new initiatives or provide comprehensive care. Finally, Bunn et al. (2016) reported that Admiral Nurses often faced challenges managing large caseloads, which impact their ability to provide optimal support.

## 5 Discussion

The global population is rapidly ageing, with the number of people over 60 projected to double to 2.1 billion by 2025 (World Health Organisation (WHO) 2024). This demographic shift is driving changes in health and social care systems worldwide. Although ageing itself is not synonymous with ill health, older adults are at increased risk of experiencing conditions such as frailty, urinary incontinence, falls and delirium (World Health Organisation (WHO) 2024). With an increased prevalence of individuals living with complex conditions, prioritising quality of life is paramount. Care models must evolve—shifting focus from acute and clinical care to greater investment in social care and community‐based support.

In this context, given the global fiscal challenges facing healthcare systems, the role of nurses, particularly ANs, becomes increasingly crucial. Although synthesising costs across international studies presents challenges (Abraham et al. 2019), evidence suggests that full scope of practice for ANs can deliver significant economic and human cost benefits (Conover and Richards 2015). The growing need for social care, coupled with the potential of ANs, brings the intersection of these two critical areas into sharp focus.

Granted, international comparisons of social care are challenging because of variations in health systems, governance, financing, and cultural contexts (Chavez et al. 2018). Plus, despite nursing being a globally present profession, this review highlights significant differences in scope, regulation and terminology across countries. The US has a well‐defined regulatory framework for APRNs, whereas Australia legally regulates only one advanced practice role—the Nurse Practitioner (Chief Nursing and Midwifery Officers Australia 2022). In contrast, the UK currently lacks cohesive formal oversight of role titles, resulting in a proliferation of skilled advanced nursing positions under various terms.

However, synthesising the available international literature does provide valuable insights. This scoping review demonstrates how ANs enhance both immediate, person‐centred care and long‐term outcomes, such as reduced medication use and hospitalisations. By strengthening team dynamics and collaboration, ANs foster more cohesive care environments. Their diverse contributions across clinical practice, education, leadership and change implementation are critical to elevating care quality and enhancing resident outcomes in social care.

This review also offers key lessons for advancing the role of ANs in social care. Their successful integration hinges on role clarity and acceptance, strong collaboration built on trust, regular supervision and organisational commitment. Granting ANs autonomy empowers them to navigate complex social care settings, optimise resident care and elevate practice standards. Collaboration is central to this discussion; it enables ANs to thrive, fosters interprofessional partnerships, and enhances care delivery.

Second, barriers to implementation are layered and need addressing. Where some are practical ‘on the ground’ issues, other institutional and policy‐level barriers, such as workforce shortages, are also deeply intertwined and work to hinder effective integration of the AN role. Additionally, the relationship between social care environments and the wider healthcare ecosystem, such as effective collaboration with General Practitioners and other professionals, plays a critical role in the success of these roles. As such, unlocking the full potential of ANs in care homes and charitable organisations should not be viewed as an isolated goal, rather framed as part of broader efforts to futureproof the sector.

These globally pertinent findings have important implications for UK policy and practice. They support the British Geriatrics Society's (BGS) 2021 statement, which emphasises the need for UK government funding to support care home initiatives that enable residents to receive care in situ that would otherwise necessitate hospital admission. Also, this research responds to calls for work to explore RNs' scope of practice and potential across ASC (Cornes and Manthorpe 2022) and to better define the competencies required for enhanced and advanced nursing practice in social care (Gavrielides et al. 2024). These findings are particularly timely, coinciding with both the recent introduction in the UK of the SPQ pathway in ASC Nursing (QNI 2022) and the NMC's ongoing review of Advanced Practice, which aims to formally regulate advanced practice education and roles (Nursing and Midwifery Council 2025).

Ultimately, this review provides a foundation for mapping current knowledge while also revealing significant gaps. First, the literature does not sufficiently capture the contributions of ANs in charitable organisations. Although Bunn et al. (2016) provide some insights, further research is needed to understand the breadth and depth of their roles in these settings. The nuanced, non‐observable elements of AN work such as interpersonal interactions, professional judgement and clinical decision‐making are also underexplored. As Lee et al. (2023) highlight, these components are critical to advanced practice and warrant further investigation. It is also notable that none of the studies included in this review specifically examined the perspectives of residents or families; understanding their experiences and views is crucial for evaluating and refining advanced nursing roles in social care settings.

To realise the full potential of AN roles in social care nursing settings, the prevailing perceptions that social care nursing is not a place for professional growth and development need challenging. Instead, such environments should be recognised as a dynamic and rewarding field for nurses to learn, innovate and advance their practice. By recognising and addressing barriers, fostering collaboration and investing in research, the contributions of ANs and their career pathways are optimised, ultimately improving care outcomes. By leveraging their expertise, ANs can contribute to more efficient and effective health and social care delivery, reducing the overall burden on acute settings.

### 5.1 Strengths and Limitations

A key strength of this review was its systematic approach to investigating advanced nursing roles in social care. Despite efforts to source grey literature, it is likely that not all was identified. Additionally, the exclusion of articles published in languages other than English represents a limitation of this review. It is also important to recognise that, given this review focused on care homes and charitable organisations, capturing or exploring the diversity of specialist services that support *adults* with a learning disability, autism, *mental health* conditions or physical disabilities was outside the scope of this review.

## 6 Conclusions

In the evolving landscape of social care, ANs play a critical role in enhancing care delivery, improving outcomes, and fostering interdisciplinary collaboration. However, the integration of ANs into social care settings requires strategic planning, role clarity and organisational support. This review highlights the economic and human cost benefits of fully utilising ANs' scope of practice. Their contributions extend beyond clinical practice to encompass leadership, education, and systemic change implementation, ultimately strengthening team dynamics and care outcomes. Yet, it is also clear that challenges remain, particularly regarding workforce shortages, inconsistent role definitions and varying regulatory frameworks across countries. We propose the following to advance understanding and support the effective utilisation of ANs in social care:

- Conduct further research to examine ANs' contributions across the diverse social care landscape. Evidence from social care settings outside of care homes, such as charitable organisations, remains very limited. Future work should also incorporate the perspectives of residents and families and address geographic gaps, including the UK.
- Establish and enhance structured pathways for professional growth, including mentorship programmes and continuing education, to attract, develop and retain ANs in social care settings.
- Encourage initiatives that investigate how ANs can strengthen integration between health and social care, including collaboration with primary, secondary, community and tertiary care services.
- Formulate a broader set of metrics to capture ANs' contributions to care quality, resident outcomes, and system efficiency across diverse social care settings.
- Develop a clearer understanding of the economic impact of AN‐led initiatives to inform policy and organisational decision‐making.

By investing in AN roles and fostering collaboration, social care can be strengthened, leading to improved outcomes for ageing populations, reduced pressure on acute care settings and a shift in perceptions regarding the professional development opportunities available in social care.

## Author Contributions

All authors have agreed on the final version and meet at least one of the following criteria: (1) substantial contributions to conception and design, acquisition of data, or analysis and interpretation of data; (2) drafting the article or revising it critically for important intellectual content.

## Conflicts of Interest

The authors declare no conflicts of interest.

## Supporting information


**Table S1:** Extraction table.

## Data Availability

The data that support the findings of this study are available from the corresponding author upon reasonable request.
