# Supplementary material for: Exploring the Presence and Impact of Advanced Nursing Roles in Care Homes and Charitable Organisations: An International Systematic Scoping Review
Source: J Adv Nurs. 2025 Sep 16;82(6):5601–14. doi: 10.1111/jan.70212 (PMC13176703; doi:10.1111/jan.70212)
Supplement: Supplementary file 1 — Table S1: Extraction table. [file JAN-82-5601-s001.docx]

Table S1: Extraction table
